# Comparison of surgical results in patients with hypertrophic obstructive cardiomyopathy after classic or modified morrow septal myectomy

**DOI:** 10.1097/MD.0000000000009371

**Published:** 2017-12-22

**Authors:** Yongqiang Lai, Hongchang Guo, Jinhua Li, Jiang Dai, Changwei Ren, Yang Wang

**Affiliations:** Department of Cardiac Surgery, Beijing Anzhen Hospital, Capital Medical University, Beijing, China.

**Keywords:** hypertrophic obstructive cardiomyopathy, myectomy

## Abstract

The study was conducted to evaluate the surgical results in patients with hypertrophic obstructive cardiomyopathy (HOCM) who underwent either classic Morrow septal myectomy or modified procedure.

The modified Morrow septal myectomy has gained interest as a new treatment for patients with drug-refractory symptoms of HOCM; however, its benefits in comparison to the classic procedure are unknown.

In all, 236 symptomatic HOCM patients underwent surgical treatment from January 2006 to January 2015. Among them, 86 patients were treated by the classic Morrow myectomy and 150 patients via the modified procedure. Septal thickness, maximal left ventricular outflow tract (LVOT) pressure gradient (PG), and the presence of a permanent pacemaker were recorded after operation and follow-up

The left ventricular septal thickness (22.1 ± 11.9 vs 17.1 ± 4.0 mm for classic procedure, and 22.3 ± 4.4 vs 16.1 ± 3.5 mm for modified procedure; *P* < .001), LVOT velocity (410.6 ± 134.0 vs 210.5 ± 81.4 mm/s for classic procedure, and 432.7 ± 119.3 vs 167.7 ± 50.1 mm/s for modified procedure; *P* < .001), LVOT PG (76.0 ± 43.5 vs 19.8 ± 16.7 mm Hg for classic procedure, and 80.8 ± 40.7 vs 12.3 ± 8.5 mm Hg for modified procedure; *P* < .001) were significantly decreased after the operation in both groups. The modified group, however, showed significantly greater reduction in these echocardiographic parameters than the classic group. PG was completely eliminated in 142 (94.7%) patients receiving the modified myectomy, and a resting PG over 30 mm Hg was demonstrated in 16 (18.6%) patients in the classic group at follow-up (*P* = .001). Thirty-two (37.2%) patients in the classic groups had a mitral valve replacement, which is significant more than 14 (9.3%) in the modified group (*P* < .001).

Both the classic procedure and the modified procedure can reduce LVOT obstruction and alleviate symptoms in patients with HOCM. The modified Morrow septal myectomy is superior to the classic procedure in reducing the LVOT gradient with a lower incidence of mitral valve replacement.

## 1 Introduction

Hypertrophic obstructive cardiomyopathy (HOCM) is a unique primary myocardial disease characterized by hypertrophy of the interventricular septum, a narrowed left ventricular outflow tract (LVOT), and, frequently, systolic anterior motion (SAM) of the mitral valve resulting in LVOT obstruction.^[1–3]^ Medical treatment is the first-line therapy for symptomatic patients with LVOT obstruction. However, left ventricular septal myectomy is recommended if medical treatment is unsuccessful or intolerable.^[4–6]^

The classic Morrow myectomy was described for the first time in 1975.^[7]^ In the classic Morrow procedure, left ventricular outflow tract obstruction is relieved by resecting relatively small sections of muscle tissue in the proximal ventricular septum, which widens left outflow tract and decreases the hydrodynamic drag forces along with a “Venturi” effect resulting in SAM.^[8]^ Thus, myectomy procedure allows a gradient reduction that can relieve clinical symptoms.^[9]^ Recently, many different modifications of the classic procedure have been reported, aiming to dredge the left ventricular outflow with better outcomes by extending the resected area.^[9–13]^ However, its benefits in comparison to the classic procedure are unknown. In this study, we compared surgical outcomes, symptom resolution, hemodynamics, and complications in patients with HOCM undergoing either the classic or the modified Morrow septal myectomy.

## 2 Materials and methods

### 2.1 Patients

In all, 236 consecutive patients with HOCM (aged >18 years) with significant LVOT obstruction were studied with the initial visit from January 2006 to January 2015 at the Department of Cardiac Surgery of Beijing Anzhen Hospital, Beijing, China. The study protocol was approved by the ethics committee of Beijing Anzhen Hospital. The following exclusion criteria included: concomitant moderate or greater aortic/mitral stenosis; maximal (including provocable) LVOT pressure gradient (PG) less than 50 mm Hg; apical hypertrophic cardiomyopathy (HCM) variant; and hypertensive heart disease in older patients.^[14–16]^ HCM was diagnosed by experienced cardiologists on the basis of typical features, with ventricular myocardial hypertrophy (left ventricular wall thickness >15 mm) in the absence of any other disease responsible for hypertrophy.^[17,18]^ Resting/provocable LVOT obstruction (LVOT gradient >50 mm Hg) were also included.

### 2.2 Clinical data collection

The clinical data and demographic information were extracted from the medical records of each patient, including demographics, clinical outcomes, and echocardiographic parameters. Complications including the need for permanent pacing between 2 groups were compared. The echocardiographic parameters were analyzed including interventricular septal thickness, left ventricular outflow gradients, left ventricular ejection fraction, degree of mitral regurgitation, and SAM of the mitral valve. Preoperative mitral valve insufficiency degree was determined by echocardiography^[19]^and categorized as mild (regurgitation jet area <4 cm^2^), moderate (regurgitation jet area >4 and <8 cm^2^), and severe (regurgitation jet area >8 cm^2^).

### 2.3 Operative technique

Standard cardiopulmonary bypass and myocardial preservation techniques were used in the both groups. A transverse aortotomy is made, carried rightward toward the noncoronary sinus, and retracted with pledgeted sutures. The aortic wall at the distal valve commissures is suspended with pledgeted sutures to maximize exposure of the hypertrophied septum and anterior mitral leaflet. Mitral valves were managed by interatrial groove pathways. Operations were guided with transesophageal echocardiography.

**Figure 1:**
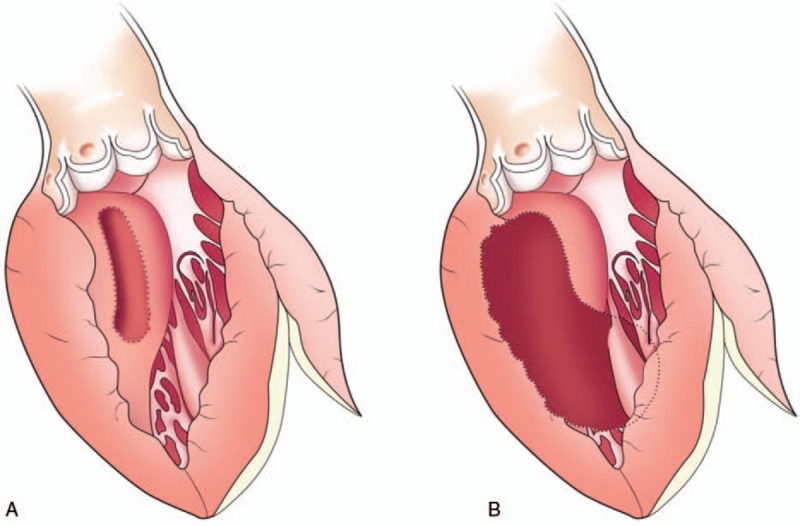
The resection areas were marked with shaded areas. (A) The classic Morrow procedure. (B) The modified procedure.

For classic Morrow septal myectomy, the resection is started by making 2 parallel longitudinal incisions in the septum, the first beneath the nadir of the right coronary cusp and the second beneath the commissure between the right and the left coronary cusps, as previously designed by Morrow.^[7]^ These incisions are connected superiorly with a third incision 1.0 to 1.5 cm below the aortic valve, and a deep wedge of septal tissue is resected (Fig. 1 A).

For modified Morrow procedure, the incision was made by extending the classic incision with a midventricular resection, beginning with continued resection leftward toward the mitral valve annulus and apically to the bases of the papillary muscles. All areas of papillary muscle fusion to the septum or ventricular free wall were divided, and anomalous chordal structures, muscle bundles, and fibrous attachments of the mitral leaflets to the ventricular septum or free wall were divided or excised (Fig. 1B).

### 2.4 Following up

Patients were followed-up at 3 and 12 months after operation, and yearly thereafter. Physical examination and echocardiography were recommended during follow-up. The follow-up was carried out by subsequent clinic visits to the outpatient departments and telephone interviews with the patients and their relatives.

### 2.5 Statistical analysis

Continuous variables are expressed as means standard deviations (SDs), and categorical variables as frequencies or percentages. SPSS V.22 (SPSS, Inc., IBM, Chicago, IL) was used for the statistical analysis. Categorical variables were compared using chi-square or Fisher exact tests. Quantitative variables were compared using the paired-samples *t* test. A *P* value of <.05 was considered significant for comparison of clinical outcomes.

## 3 Results

### 3.1 Baseline clinical profiles

Of the 236 HOCM patients, 86 (36.4%) patients were treated with classic Morrow myectomy and 150 (63.6%) with modified Morrow procedure. A significantly higher proportion of patients had syncope as a presenting feature in the modified group compared with the classic group (23.3% vs 56%; *P* < .001). No significant differences of other baseline clinical and echocardiographic profiles were observed between the 2 groups (Table 1).

**Table 1:**
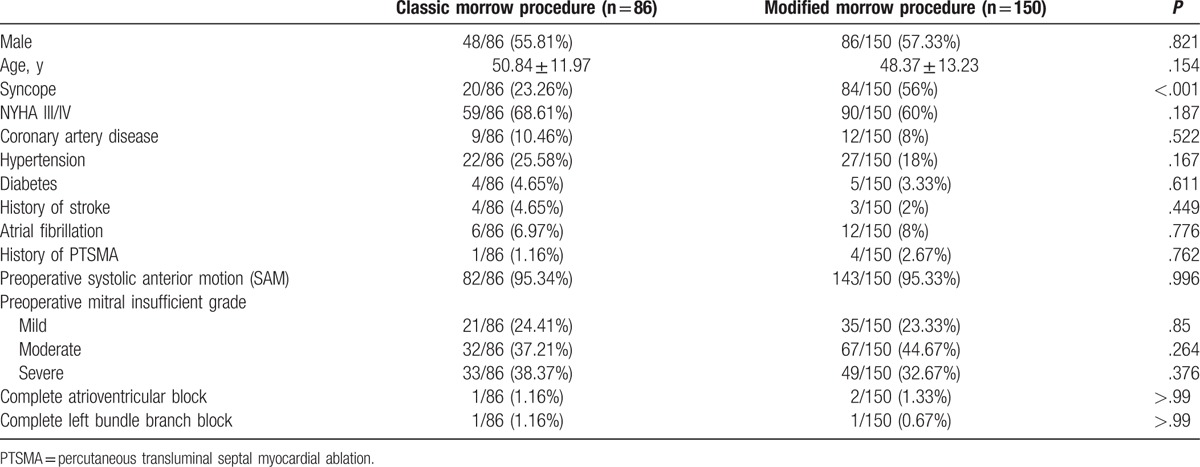
Baseline characteristics of the 2 groups.

### 3.2 Echocardiographic outcomes

The ejection fraction pre and postprocedures were within normal ranges for both groups (Table 2). The mean left ventricular outflow gradient decreased from (76.1 ± 43.5) mm Hg to (19.8 ± 16.7) mm Hg in the classic group (*P* < .001), and from (80.8 ± 40.7) mm Hg to (12.3 ± 8.5) mm Hg in the modified group (*P* < .001), indicating a significant hemodynamic improvement with both procedures. The residual resting PGs were significantly lower in the modified group than in the classic group after operation. PG was completely eliminated in 142 (94.7%) patients with modified myectomy, whereas 16 (18.6%) patients with classic procedure still demonstrated a resting PG over 30 mm Hg at follow-up (*P* < .001). The septal thickness reduced from (22.1 ± 11.9) to (17.1 ± 4.0) mm in the classic group (*P* < .001), and from (22.3 ± 4.4) to (16.1 ± 3.5) mm (*P* < .001) in the modified group. There was a significant difference in the absence in SAM between 2 groups. SAM disappeared from 95.4% to 14.0% in classic group, and from 95.3% to 4.7% in modified group.

**Table 2:**
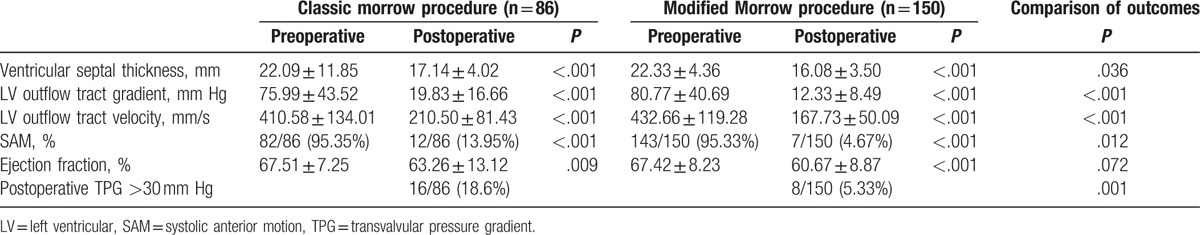
Clinical outcomes and hemodynamic result.

### 3.3 Procedural, clinical outcomes, and follow-up

There was no significant difference in cross-clamp time (102.0 ± 45.9 minutes in classic group and 94.3 ± 54.3 minutes in modified group; *P* = .267), and amount of blood loss (879.7 ± 375.1 mL in classic group and 836.4 ± 290.0 mL in modified group; *P* = .324). Thirty-two (37.2%) patients need mitral valve replacement (MVR) in the classic group, whereas only 14 (9.3%) patients in the modified group need MVR (*P* < .001). There was no difference in the proportion of mitral valvoplasty, and the mean duration of intensive care unit hospitalization between 2 groups. Two patients in modified group needed extracorporeal membrane oxygenation for postoperative low cardiac output. Twenty-five (29.1%) patients in the classic group had complete left bundle branch block, compared with 59 (39.3%) patients in the modified group (*P* = .274). Permanent pacemakers due to complete heart block were required in 4 patients (4.7%) of the classic group and in 2 patients (1.3%) of the modified group, respectively (*P* = .259).

The mean follow-up was 33.5 ± 24.7 months for the classic group and 36.7 ± 25.0 months for the modified group. Six (6.9%) patients in the classic group and 5 (3.3%) patients in the modified group were lost to long-term follow-up. Two patients in the classic group died in the early postoperative period (<1 month), 1 from sepsis and another from progressive cardiac failure. Two patients died of low cardiac output in the modified Morrow group. There was 1 late death (>12 months) in the classic group; the patient died of stoke caused by endocarditis. There was no significant difference between groups in survivals. Kaplan-Meier curves demonstrated no difference in long-term survival between classic and modified morrow septal myectomy (log-rank *P* value = .268). Two patients received redo-MVR, 1 in the classic group for severe mitral valve regurgitation who underwent mitral valvoplasty before, and the 1 in the modified group from mitral valve vegetation caused by endocarditis. Echocardiography examination demonstrated the modified group had a lower LVOT PG at 1 year after the operation (Table 3). Seven (9%) patients were evaluated for NYHA III/IV in the classic group, which was significant more than 3 (2%) in the modified group at the latest follow-up.

**Table 3:**
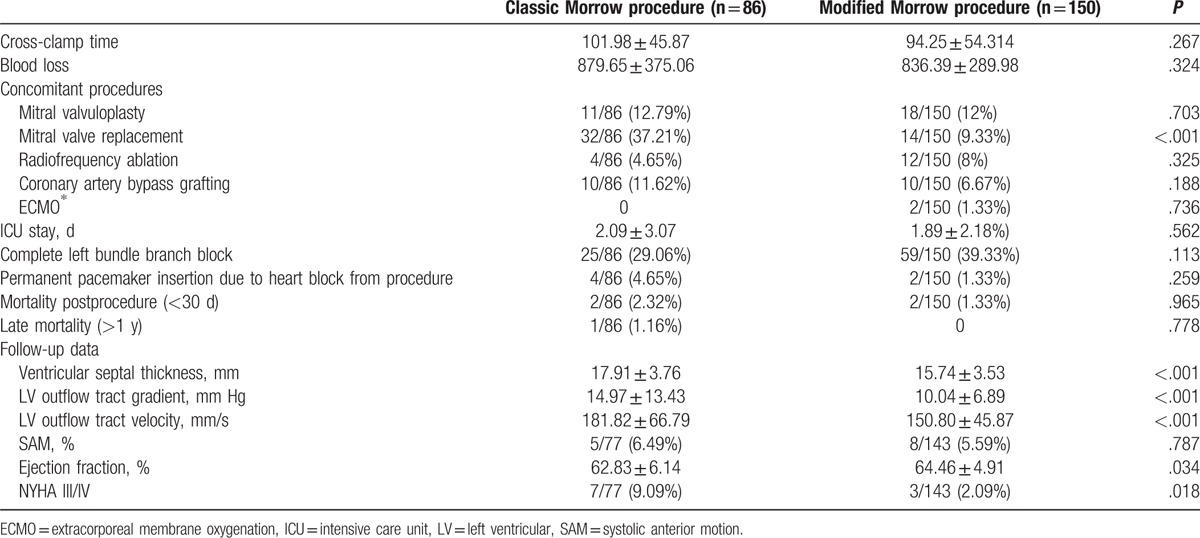
Complications and follow-up data.

## 4 Discussion

Hypertrophic cardiomyopathy is characterized by inappropriate hypertrophy of the myocardium and is associated with various clinical presentations ranging from complete absence of symptoms to sudden, unexpected death. Generally, the basal septum hypertrophy and SAM of the mitral valve are 2 important components to cause LVOT obstruction. SAM of the mitral valve could result in mitral-septal apposition and incomplete leaflet apposition. Operation has been proved to be the gold standard therapy for those severely symptomatic patients with fixed or inducible gradients who are intolerant of these medications or unresponsive to them.^[20]^

Myectomy procedures had been first reported by Morrow in 1975. Many variations of this procedure had been reported with varied efficacies after then.^[10,12]^ Wang et al^[21]^ and Gao et al^[13]^ reported modified Morrow procedure could improve or eliminate SAM, which is consistent with the findings of our study. Similarly, Minakata et al^[12]^ reported good early results in HOCM patients after extended septal myectomy with significantly decreased mortality and morbidity, even in high-risk patients with severe symptoms. In our study, the classic procedure could alleviate left ventricular outflow PG with acceptable result—the patients with modified procedure had a lower left ventricular outflow PG with an extended septal resection postoperatively. Complete left bundle block was similar in 2 groups; permanent pacemaker insertion due to complete heart block was relatively higher in classic group, although significant difference was not demonstrated in statistics. Among patients with the modified Morrow procedure, 98% were asymptomatic or only showed mild dyspnea during follow-up. Therefore, the modified Morrow procedure demonstrated optimal results compared with the classic surgery, with potential long-term survival benefits, immediate alleviation of symptoms, and the incidence of complications.

Management of mitral valve is a big challenge to HOCM surgery. About 66% of HOCM patients have structural abnormalities of the mitral valve, including enlarged leaflet area, leaflet elongation, or anomalous papillary muscle insertion into the anterior mitral leaflets. These anomalies can lead to residual obstruction if not recognized and managed.^[22]^ Papillary muscle fusion connection should be separated, and isolation is required in case of abnormal chordae tendineae or mitral valve fiber attachment to the ventricular septum or left ventricular free wall during surgery. Wider resection with the modified Morrow procedure may have better clinic outcome for treating papillary muscle connection and fusion to the septum, achieving long-term symptom alleviation in a large portion of patients.^[23]^ Previous studies from experienced surgeons have shown that, in the absence of iatrogenic mitral valve injury, pre-existing mitral valve prolapse, or chordal rupture, mitral valve surgery is not needed as long as the myectomy is adequate.^[24]^ Extensive myectomy results in enlargement of the LVOT area and redirection of forward flow with loss of the drag and Venturi effects on the mitral valve. Mitral valve replacement is only recommended for patients with mitral valve structural abnormalities, such as iatrogenic mitral valve injury, pre-existing mitral valve prolapse, or chordal rupture. Although there was no significant difference in mitral valvoplasty between 2 groups, 37.2% patients need MVR in the classic group, comparing with 9.3% patients in the modified group in our study. The proportion of MVR was lower in the modified group. Inadequate myectomy and mitral valve structural abnormalities may be the main reasons for the higher incidence of MVR in the classic group. MVR, however, can eliminate symptoms of SAM at the cost of increasing the risk of severe artificial valve-related complications.^[25]^

### 4.1 Study limitations

This study was not a randomized trial. Different surgeons may adopt either the classic procedure or the modified procedure according to their experiences. There were no criteria for dividing patients into classic or modified groups.

## 5 Conclusions

In conclusion, both the classic procedure and the modified procedure can reduce LVOT obstruction and alleviate symptoms in patients with HOCM. The modified Morrow myectomy is superior to the classic procedure in reducing the left ventricular outflow tract gradient with a lower incidence of MVR.
